# Environmental Health Oriented Optimal Temperature Control for Refrigeration Systems Based on a Fruit Fly Intelligent Algorithm

**DOI:** 10.3390/ijerph15122865

**Published:** 2018-12-14

**Authors:** Yuxiao Qin, Li Sun, Qingsong Hua

**Affiliations:** 1 Jiangsu Province Key Lab of Aerospace Power System, Chien-Shiung Wu College, Southeast University, Nanjing 210096, China; 213151403@seu.edu.cn; 2 Key Lab of Energy Thermal Conversion and Control of Ministry of Education, Southeast University, Nanjing 210096, China; 3 School of Mechanical and Electrical Engineering, Qingdao University, Ningxia Road 308, Qingdao 266071, China

**Keywords:** environmental health, energy saving, refrigeration system, fruit fly optimization algorithm (FOA)

## Abstract

The recent decades have witnessed refrigeration systems playing an important role in the life of human beings, with wide applications in various fields, including building comfort, food storage, food transportation and the medical special care units. However, if the temperature is not controlled well, it will lead to many harmful public health effects, such as the human being catching colds, food spoilage and harm to the recovering patients. Besides, refrigeration systems consume a significant portion of the whole society’s electricity usage, which consequently contributes a considerable amount of carbon emissions into the public environment. In order to protect human health and improve the energy efficiency, an optimal control strategy is designed in this paper with the following steps: (1) identifying the refrigeration system model based on a least squares method; (2) tuning an initial group of parameters of the proportional-integral-derivative (PID) controller via the pidTuner Toolbox of Matlab; (3) using an intelligent algorithm, namely fruit fly optimization (FOA), to further optimize the parameters of the PID controller. By comparing the optimal PID controller and the controller provided in the reference, the simulation results demonstrate that the proposed optimal PID controller can produce a more controllable temperature, with less tacking overshoot, less settling time, and more stable performance under a constant set-point.

## 1. Introduction

Nowadays, refrigeration systems play an essential part in the daily life of human beings. Controlling the temperature by refrigeration techniques is involved in various areas such as human comfort, food storage, food transportation and the environment [1]. However, the systems have to work in the manner of moving the heat from a cold reservoir to a hot reservoir [2], requiring high energy consumption. With the development of the society and the urbanization process, refrigeration techniques are applied everywhere, which causes an acceleration of the growth of the carbon emissions around the world [3]. According to the surveys, almost 30% of the energy consumed all over the world is utilized for Heating, Ventilating, and Air Conditioning (HVAC) [4], while refrigerators account for about 28% of all the energy consumption of a typical US family [5].

Owing to the fact that refrigeration systems are closed cycles, their elements are connected with diverse valves and pipes, which leads to a strong nonlinearity [2], which adds to the dynamic modeling difficulties. Plus, the coupling characteristics increase the difficulty of the design of a controller for the system. All these characteristics of refrigeration systems make their temperature control challenging, and inefficient control strategies may even cause the problem of temperature fluctuation. When the temperature of the environment where people live varies from time to time, it’s very likely for those people to get ill, and this is bad for public health.

To solve the problem of saving energy and improving the refrigeration effect, efficient control strategies are of great importance [6]. Several methods have been used for the control of refrigeration systems. In Ma’s and Bayram’s studies [7,8], fuzzy logic control was applied to control the temperature of a refrigeration system, while in Pedersen’s study [9], a neural network is combined with a gain scheduling-based PI controller to control the overheating of a refrigeration system. In addition, in Yin’s and Schalbart’s studies [10,11], MPC controllers are utilized to control refrigeration systems, and a L-Band SBQP-Based MPC control scheme has also been applied to control two different devices in a supermarket refrigeration system [12]. Although all these control strategies achieve a satisfactory control performance, they have the same disadvantage, which is complexity. For example, to implement a MPC scheme, a great amount of calculation is required, which needs to be performed by a high-performance computer, and this makes the application of MPC schemes hard to realize [13]. 

Proportional-Integral-Derivative (PID) controllers have been widely used in industrial applications for a long time for their simple structures, accuracy and degree of stability performance [14], thus they can also be used for the control of refrigeration systems. Generally speaking, there are two ways to tune a PID controller, which are analytically and numerically [15]. However, due to the coupling of the refrigeration system, the controller consists of two PID controllers and this constitutes a MIMO process. As a result, there will be six parameters that need to be determined, which makes the controller hard to tune. 

The fruit fly optimization algorithm (FOA), proposed by Pan in 2011 [16], is a kind of stochastic optimization algorithm that selects a result with certain rules. With the merits of fast convergence and easy programmability, it is widely used to solve optimization problems [17,18,19]. To this end, a fruit fly optimization algorithm is applied to tune and optimize the parameters of a refrigeration controller in this paper. The position of each fruit fly stands for a set of parameters of the controller, and with iteration, the fly swarm will finally arrive at a location with the best smell concentration [20], which represents the set of parameters possessing the best control performance.

To summarize, this paper: (1) identified the transfer function the refrigeration system model; (2) uses FOA to optimize the parameters of the PID controller based on the identified model; (3) uses the optimal PID controller to control the refrigeration system model. This paper is organized as follows: in Section 2, the model of the refrigeration system is described and the control problems are analyzed, while in Section 3, the transfer function of the refrigeration system is identified, the RGA paring is done to analyze the relationship between the system variables, and the parameters of the PID controller for the identified model are optimized. In Section 4, the optimal PID controller is put into use to control the refrigeration system, and conclusions are drawn in Section 5.

## 2. System Description

### 2.1. Model Description

**Figure 1:**
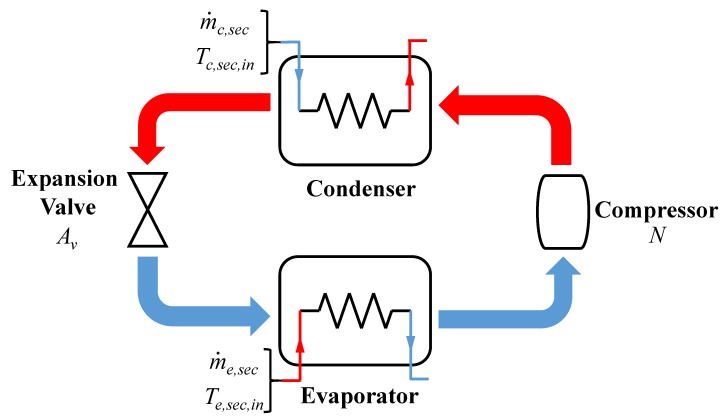
The composition of the refrigeration system.

The refrigeration system model in this paper, based on vapor compression, is proposed in Bejarano’s study [2]. The system consists of a compressor, a condenser, an expansion valve, and an evaporator, and the composition of the system is shown in Figure 1 [2]. The goal of this system is to remove the heat in the secondary flux of the evaporator and then deliver it to the secondary flux of the condenser. The system is based on an inverse Rankine cycle, and it works as follows: firstly, the refrigerant flows through the evaporator under low temperature and low pressure conditions. In this way, the heat in the evaporator secondary flux is removed. Then, the temperature and the pressure of the refrigerant are increased with the help of the compressor, and then it enters the condenser. The temperature of the refrigerant decreases after it flows through the condenser, and during this process, it may become a sub-cooled liquid. Finally the pressure and the temperature of the refrigerant decrease again after flowing through the expansion valve, and the next round of the cycle will start once it enters the evaporator.

As illustrated in Figure 1, this system is a MIMO system, where by manipulating the two variables *A_v_* (the opening percentage of the expansion valve) and *N* (the speed of the compressor), another two variables *T_e,sec,out_* (the outlet temperature of the evaporator secondary flux) and *T_SH_* (the degree of superheating) are controlled. All the other variables are the disturbances of the system. The range and the initial values of the variables in the system are displayed in Table 1, according to [2].

**Figure 2:**
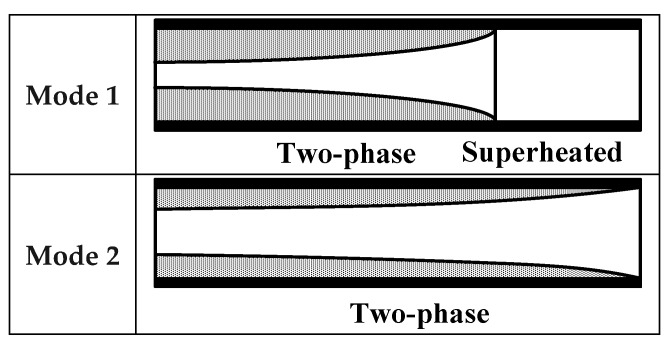
Modes of the evaporator.

**Figure 3:**
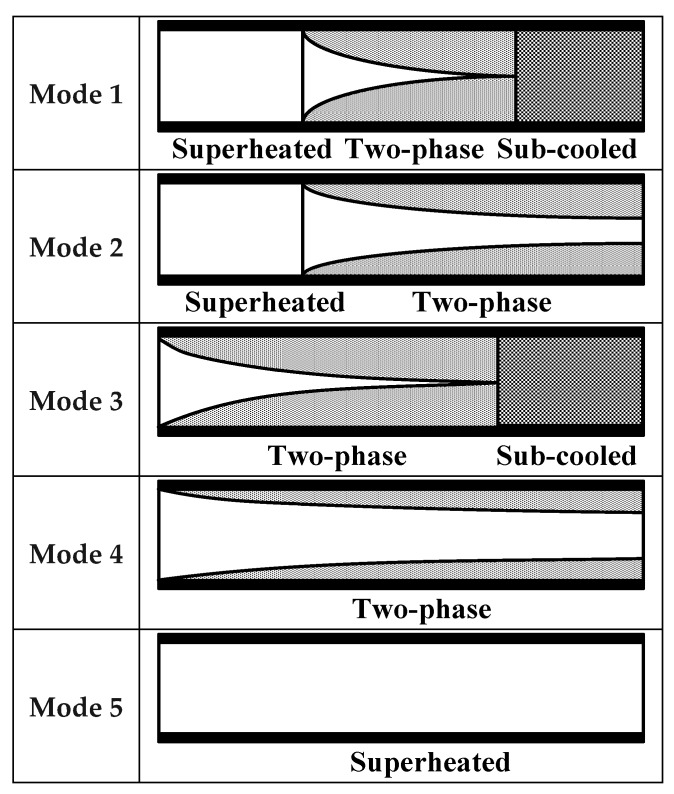
Modes of the condenser.

The model of the system is established by the switched moving boundary (SMB) method, which was first proposed by McKinley and Alleyne in 2008 [21]. With different conditions of the fluid in the heat exchanger, the model can be classified into different modes. As for the evaporator, the modes are classified by the amount of superheated steam as is shown in Figure 2, and the conditions of the condenser are categorized into five modes as illustrated in Figure 3 [22].

### 2.2. Control Problems

The goal of the control design is to maintain the outlet temperature of the evaporator secondary flux *T_e,sec,out_* and the degree of superheating *T_SH_* following their reference as accurately as possible. The problems to designing the control strategy are listed below:

- Strong nonlinearity: Owing to the fact that the refrigeration system is a closed cycle, its elements are connected with diverse valves and pipes, this leads to the result of strong nonlinearity, which adds to the difficulty of dynamic modeling.
- High coupling: This makes the design of controller for this system complicated and challenging.
- Frequent disturbance: This requires the controller to have high robustness and be able to control the system efficiently and accurately to restrain the effect of the disturbance.
- Constrained control variables: The control variables in this paper is the condenser speed and the valve opening, and they are constrained between 30 Hz~50 Hz and 10~100%, respectively, and this may cause the problem of controller saturation.

## 3. Control Design

Owing to the fact that the refrigeration system is a nonlinear system, it’s hard to tune the parameters of the controller with the original model. Thus, in this section, the refrigeration system is identified as a linear model, and two PID controllers will be designed based on the identified model. Fruit fly optimization algorithm (FOA) is used to optimize the parameters of the controller.

### 3.1. Transfer Function Identification

**Figure 4:**
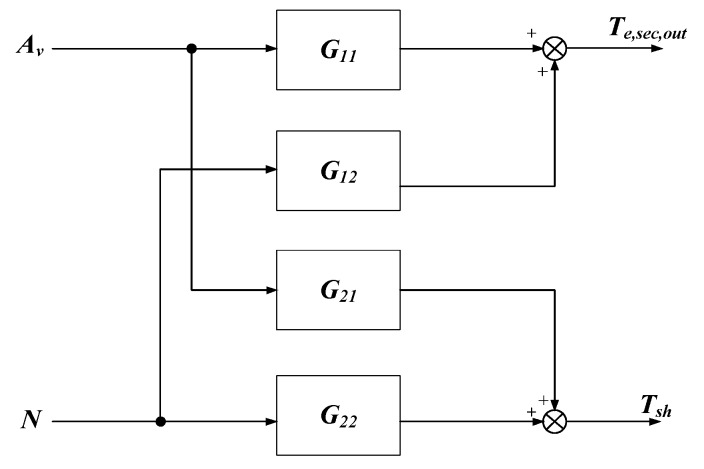
The structure of the linearized model.

**Figure 5:**
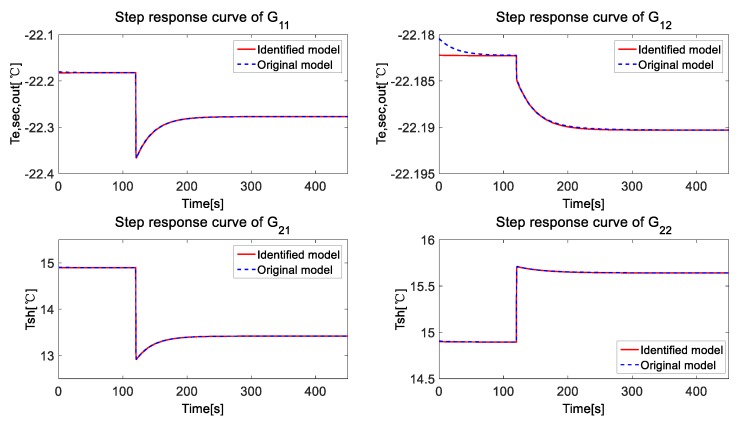
Comparison of the step response curves of the identified model and the original model.

As illustrated in Figure 1, this system is a 2 × 2 MIMO system, where by manipulating the two variables *A_v_* (the opening percentage of the expansion valve) and *N* (the speed of the compressor), another two variables *T_e,sec,out_* (the outlet temperature of the evaporator secondary flux) and *T_SH_* (the degree of superheating) are controlled. As a result, it can be described as a model with two inputs, two outputs, and four transfer functions. The structure of the identified model is shown in Figure 4. *G*_11_ presents the transfer function from *A_v_* to *T_e,sec,out_*, *G*_21_ represents the transfer function from *A_v_* to *T_SH_*, *G*_12_ presents the transfer function from *N* to *T_e,sec,out_*, *G*_22_ represents the transfer function from *N* to *T_SH_*. The process of system identification is as follows:

Firstly, a step signal is set on *A_v_*, while *N* is kept as a constant, and the step response of *T_e,sec,out_* and *T_SH_* are obtained. Then, a step signal is set on *N*, while keeping *A_v_* as a constant to obtain the system response of *T_e,sec,out_* and *T_SH_*. Finally, the step response curves are analyzed by the System Identification Toolbox of Matlab to identify the system. The comparison of the step response curves is drawn in Figure 5, and we get the transfer functions as follows:

(1) $$\begin{array}{l} G_{11}=\frac{-2.501s-0.4769}{s^{2}+67.23s+25.15}\\ G_{12}=\frac{-0.003443s-0.0003653}{s^{2}+6.699s+0.2273}\\ G_{21}=\frac{-2.713s-0.07687}{s^{2}+6.828s+0.2601}\\ G_{22}=\frac{1.145s+0.0249}{s^{2}+7.046s+0.1668} \end{array}$$

For all the identified transfer function, the fitness values are all more than 99%. As a result, the identified model can be an ideal linearized model for the refrigeration system.

### 3.2. RGA Paring

The relative gain array (RGA) which is recommended in [23], is a helpful tool for analyzing the interaction of the variables in a system. Setting *s* to 0, we get the steady state matrix of *G*_11_, *G*_21_, *G*_12_ and *G*_22_ in (2):

(2) $$A=\left[\begin{array}{cc} G_{11}|{}_{s\to 0} & G_{12}|{}_{s\to 0}\\ G_{21}|{}_{s\to 0} & G_{22}|{}_{s\to 0} \end{array}\right]=\left[\begin{array}{cc} -0.0190 & -0.0016\\ -0.2955 & 0.1493 \end{array}\right]$$

and the RGA matrix is calculated by (3):

(3) $$RGA=A\cdot {\left(A^{-1}\right)}^{T}$$

For the identified system, the RGA matrix is given as (4):

(4) $$RGA=\left[\begin{array}{cc} \lambda_{11} & \lambda_{12}\\ \lambda_{21} & \lambda_{22} \end{array}\right]=\left[\begin{array}{cc} 0.8563 & 0.1437\\ 0.1437 & 0.8563 \end{array}\right]$$

According to [23,24], the bigger the RGA matrix element is, the stronger the relationship between the system input and output. As is shown in (4), the RGA elements *λ*_11_ and *λ*_22_ are much larger than *λ*_12_ and *λ*_21_, this indicates that there is a stronger relationship between *A_v_* and *T_e,sec,out_* and between *N* and *T_SH_*. As a result, *T_e,sec,out_* can be controlled by *A_v_* easily, and *T_SH_* can be controlled by *N* easily.

### 3.3. Controller Design

With the merits of simplicity and reliability, PID controllers are still widely used for industrial process control [25,26,27]. The control equation of a PID controller is shown in (5):

(5) $$u\left(t\right)=K_{p}e\left(t\right)+K_{i}\int e\left(t\right)+K_{d}\frac{de\left(t\right)}{dt}$$

where *u*(*t*) is the control action, *K_p_*, *K_i_*, *K_d_* are the proportional, integral and derivative gain respectively, and *e*(*t*) is the tracking error.

**Figure 6:**
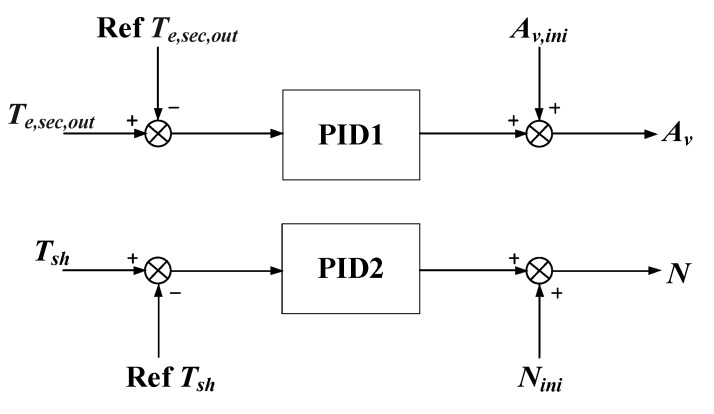
The structure of the controller.

For the high coupling of the refrigeration system, two PID controllers are applied to control the evaporator secondary flux *T_e,sec,out_* and the degree of superheating *T_SH_*, respectively, and the structure of the controller is shown in Figure 6. 

*A_v,ini_* and *N_ini_* are two constants, and they are set at 50 and 40 respectively, which are at the middle of the range of *A_v_* and *N*. Among the controller, there are six parameters we need to decide: the proportional, integral and derivative gain *K_p_*_1_, *K_i_*_1_, *K_d_*_1_ for PID1, and the proportional, integral and derivative gain *K_p_*_2_, *K_i_*_2_, *K_d_*_2_ for PID2.

We use pidTuner Toolbox of Matlab to tune the controller initially, and the initial values of the parameters of the controllers are shown as follows:

(6) $$\left\{ \begin{array}{l} K_{p1}=-20.03\\ K_{i1}=-15.21\\ K_{d1}=-0.05 \end{array}\right.,\left\{ \begin{array}{l} K_{p2}=5.14\\ K_{i2}=0.21\\ K_{d2}=0.13 \end{array}\right.$$

### 3.4. Controller Optimization

#### 3.4.1. Introduction of Fruit Fly Optimization Algorithm (FOA)

Fruit fly optimization algorithm (FOA) is a new method for solving optimization problems which was proposed by Pan [16]. This algorithm is based on the behavior of fruit fly foraging, and the parameters we aim to optimize are set as the position of the fruit fly swarm. The algorithm is executed as follows:

Firstly, the position of the fruit fly swarm is initialized randomly. After that, the direction and distance of the movement of each fly is assigned, and the position of the fly swarm is updated. Then, with the calculation result of the judgment function, the fly with the best position is determined, and all the other flies will gather together in this place. Afterwards, we assign the direction and distance of the movement of each fly again, and repeat the process, and the fly swarm will finally reach to the place nearest to the food, in other words, the parameters are optimized.

#### 3.4.2. Tuning of PID Controllers Based on FOA

**Figure 7:**
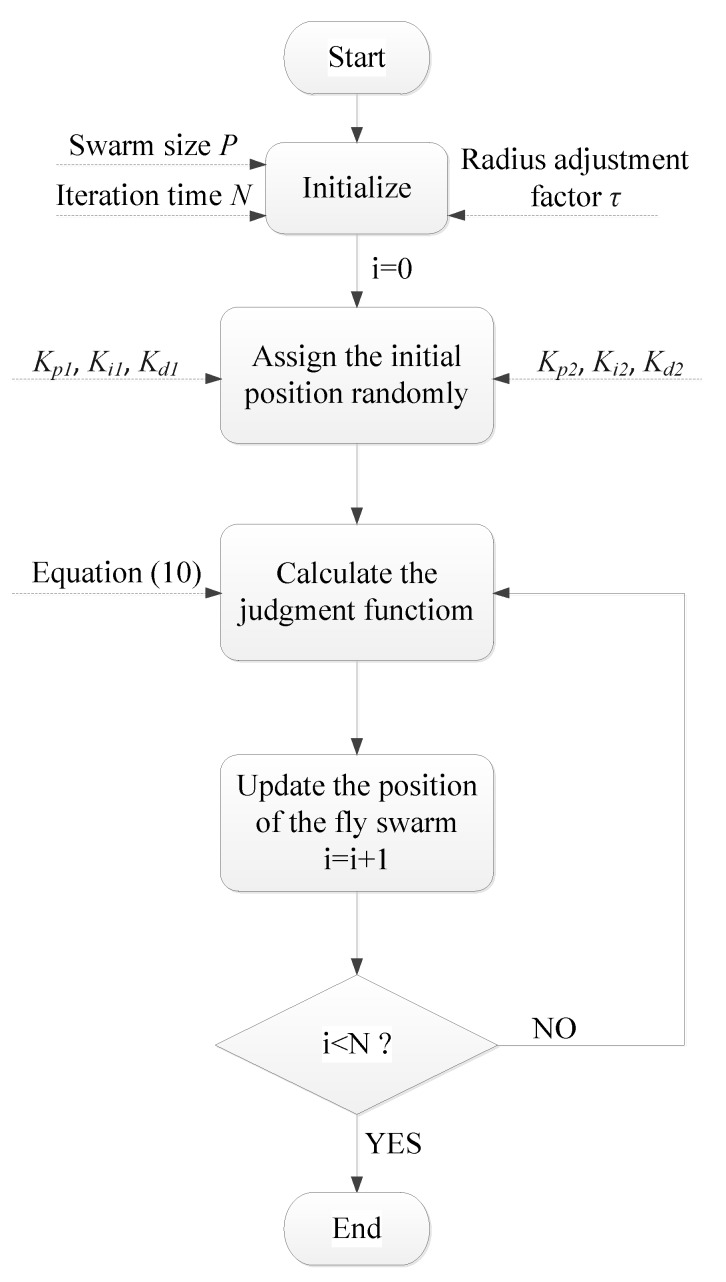
The block diagram of the FOA scheme.

In this paper, *K_p_*_1_, *K_i_*_1_, *K_d_*_1_, *K_p_*_2_, *K_i_*_2_ and *K_d_*_2_ are set as the parameters of the position of an individual fly, and the block diagram of FOA applied in this paper is drawn in Figure 7. We set swarm size *P* = 20, and the number of iterations *N* = 100, respectively. To achieve this scheme, a *P* × 6 matrix *X* in (7) is applied to represent the position of the fly swarm, and each position of the flies represents a candidate solution of the PID controller parameters.

(7) $$X=\left[\begin{array}{cccccc} K_{p11} & K_{i11} & K_{d11} & K_{p21} & K_{i21} & K_{d21}\\ K_{p}{}_{12} & K_{i12} & K_{d12} & K_{p22} & K_{i22} & K_{d22}\\ \vdots & \vdots & \vdots & \vdots & \vdots & \vdots \\ K_{p1j} & K_{i1j} & K_{d1j} & K_{p2j} & K_{i2j} & K_{d2j}\\ \vdots & \vdots & \vdots & \vdots & \vdots & \vdots \\ K_{p1P} & K_{i1P} & K_{d1P} & K_{p2P} & K_{i2P} & K_{d2P} \end{array}\right]$$

The initial position of the fly swarm is set as *K_p_*_1_ = −20.03, *K_i_*_1_ = −15.21, *K_d_*_1_ = −0.05, *K_p_*_2_ = 5.14, *K_i_*_2_ = 0.21, *K_d_*_2_ = 0.13. In each round, the position of each fly will change in a random direction and distance. The process can be achieved by the calculation of matrix in (8):

(8) $$X_{i}=\left[\begin{array}{ccc} K_{p11} & \cdots & K_{d21}\\ \vdots & \ddots & \vdots \\ K_{p1P} & \cdots & K_{d2P} \end{array}\right]+R\times \left[\begin{array}{ccc} 2\times \mathrm{rand}()-1 & \cdots & 2\times \mathrm{rand}()-1\\ \vdots & \ddots & \vdots \\ 2\times \mathrm{rand}()-1 & \cdots & 2\times \mathrm{rand}()-1 \end{array}\right]$$

where *R* is a *P* × 6 matrix indicating the radius of the range each parameter is able to change, and rand() is a random number between 0 and 1. In order to make the movable range larger at the beginning to enable the flies to get to the best position earlier, and smaller in the end to guarantee the accuracy, we make *R* vary with the iteration time. The calculation of *R* is shown in (9):

(9) $$R=\tau^{i}\times R_{0}=\tau^{i}\times \left[\begin{array}{cccccc} 2 & 1.5 & 0.005 & 0.5 & 0.02 & 0.01\\ \vdots & \vdots & \vdots & \vdots & \vdots & \vdots \\ 2 & 1.5 & 0.005 & 0.5 & 0.02 & 0.01 \end{array}\right]$$

where *R*_0_ is a *P* × 6 matrix of the initial number of the radius, *τ* is the radios adjustment factor which is between 0 and 1, and *i* is the iteration time. Here we set *τ* = 0.97, and the value of *R*_0_ is shown in (9).

With the position of each fly as the parameters of the controller, we are able to get the dynamic performance of the controller, and calculate the judgment function. We take the control accuracy, the overshoot, and the saturation time into consideration to value the control performance, and we define the judgment function as follows:

(10) $$\left\{ \begin{array}{l} J=\int_{0}^{\infty }\left(\omega_{1}t\left|e_{1}\left(t\right)\right|+\omega_{2}u_{1}{}^{2}\left(t\right)\right)dt+\alpha \int_{0}^{\infty }\left(\omega_{3}t\left|e_{2}\left(t\right)\right|+\omega_{4}u_{2}{}^{2}\left(t\right)\right)dt\\ e\left(t\right)<0,u_{1}\left(t\right)=10\mathrm{or}100,u_{2}\left(t\right)=30\mathrm{or}50\\ J=\int_{0}^{\infty }\left(\omega_{1}t\left|e_{1}\left(t\right)\right|+\omega_{2}u_{1}{}^{2}\left(t\right)+\omega_{5}\left|e_{1}\left(t\right)\right|\right)dt+\alpha \int_{0}^{\infty }\left(\omega_{3}t\left|e_{2}\left(t\right)\right|+\omega_{4}u_{2}{}^{2}\left(t\right)+\omega_{6}\left|e_{1}\left(t\right)\right|\right)dt\\ e\left(t\right)\geq 0,u_{1}\left(t\right)=10\mathrm{or}100,u_{2}\left(t\right)=30\mathrm{or}50\\ J=\int_{0}^{\infty }\left(\omega_{1}t\left|e_{1}\left(t\right)\right|\right)dt+\alpha \int_{0}^{\infty }\left(\omega_{3}t\left|e_{2}\left(t\right)\right|\right)dt\\ e\left(t\right)<0,u_{1}\left(t\right)\in \left(10,100\right),u_{2}\left(t\right)\in \left(30,50\right)\\ J=\int_{0}^{\infty }\left(\omega_{1}t\left|e_{1}\left(t\right)\right|+\omega_{5}\left|e_{1}\left(t\right)\right|\right)dt+\alpha \int_{0}^{\infty }\left(\omega_{3}t\left|e_{2}\left(t\right)\right|+\omega_{6}\left|e_{1}\left(t\right)\right|\right)dt\\ e\left(t\right)\geq 0,u_{1}\left(t\right)\in \left(10,100\right),u_{2}\left(t\right)\in \left(30,50\right) \end{array}\right.$$

where *e*_1_(*t*) and *e*_2_(*t*) are the input of PID1 and PID2, respectively, and *u*_1_(*t*) and *u*_2_(*t*) are the output of PID1 and PID2, respectively. *ω*_1_, *ω*_2_, *ω*_3_, *ω*_4_, *ω*_5_ and *ω*_6_ are the combined weights, and *α* is the adjustment factor. We set *ω*_1_ = 0.7, *ω*_2_ = 0.01, *ω*_3_ = 300, *ω*_4_ = 1.4, *ω*_5_ = 0.01 and *ω*_6_ = 600, respectively, and *α* = 0.02.

Among all the results, we pick out the position vector with the lowest judgment value, and the assignment of the positions in the next round will be based on this. After hundreds of iteration, the fly swarm will reach to the best position, and the parameters will be tuned.

#### 3.4.3. Optimization Result

We applied the identified model in 3.1 to execute FOA, and we put step signals to make *T_e,sec,out_* change from −22.15 °C to −22.65 °C at 120 s, and make *T_sh_* change from 14.65 °C to 7.2 °C at 120 s, respectively. After 100 times of iteration, we finally get the optimized parameters in (11):

(11) $$\left\{ \begin{array}{l} K_{p1}=-28.5713\\ K_{i1}=-45.5881\\ K_{d1}=-0.0628 \end{array}\right.,\left\{ \begin{array}{l} K_{p2}=6.2512\\ K_{i2}=1.3759\\ K_{d2}=0.0669 \end{array}\right.$$

**Figure 8:**
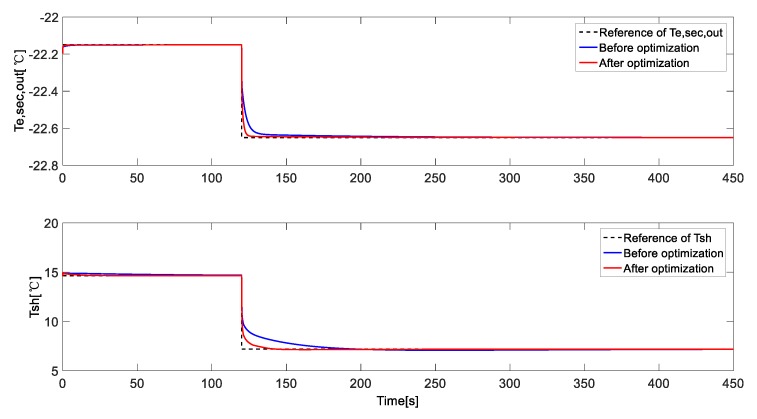
The comparison of the control performance of the controller before & after optimization.

**Figure 9:**
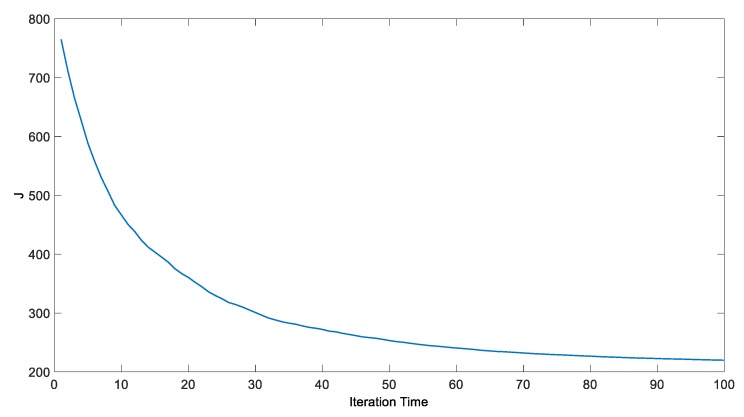
The trend line of the judgment value.

**Figure 10:**
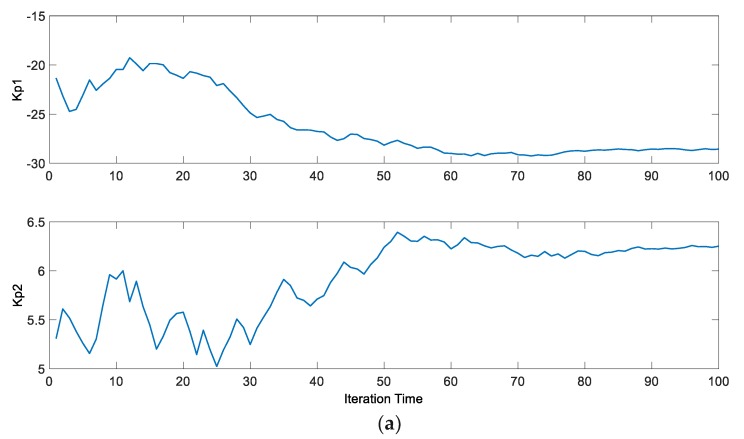

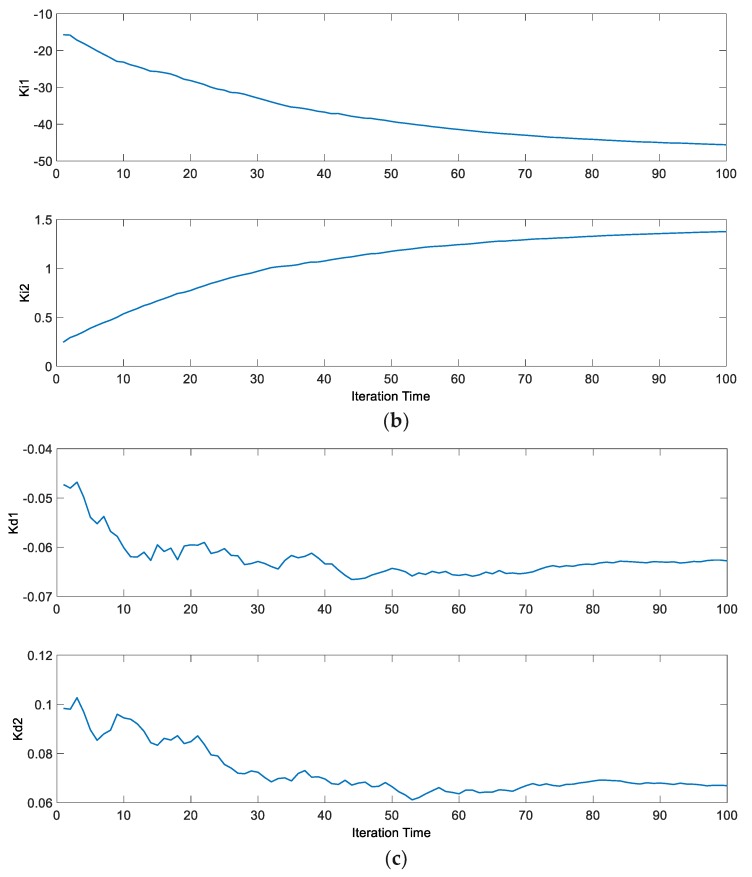
The trend curves of the parameters of the controller: (**a**) the trend curves of *K_p_*_1_ and *K_p_*_2_; (**b**) the trend curves of *K_i_*_1_ and *K_i_*_2_; (**c**) the trend curves of *K_d_*_1_ and *K_d_*_2_.

The comparison of the step response curves is shown in Figure 8, and the trend curves of the change of judgment value and each parameter are shown in Figure 9 and Figure 10, respectively.

As we can tell from the result, the optimization accelerates the control action of the controller, and reduces the settling time. The judgment value decreases from 765 to 220, and converges at about 220 after 85 iterations, *K_p_*_1_ swings lower and settles at about −28.5 after 80 iterations, and *K_p_*_2_ swings higher and settles at about 6.25 in the end. *K_i_*_1_ keeps decreasing and finally reaches about −45.5, while *K_i_*_2_ keeps increasing to reach about 1.37 at the end. *K_d_*_1_ decreases from about 0.05 to 0.063, while *K_d_*_2_ decreases from about 0.1 to 0.067.

## 4. Nonlinear Simulation

In this section, to show the robustness of the proposed controller, simulation of step response based on the proposed controller and the original controller in [2] is implemented, and the result is compared and analyzed.

### 4.1. Simulation Result

**Figure 11:**
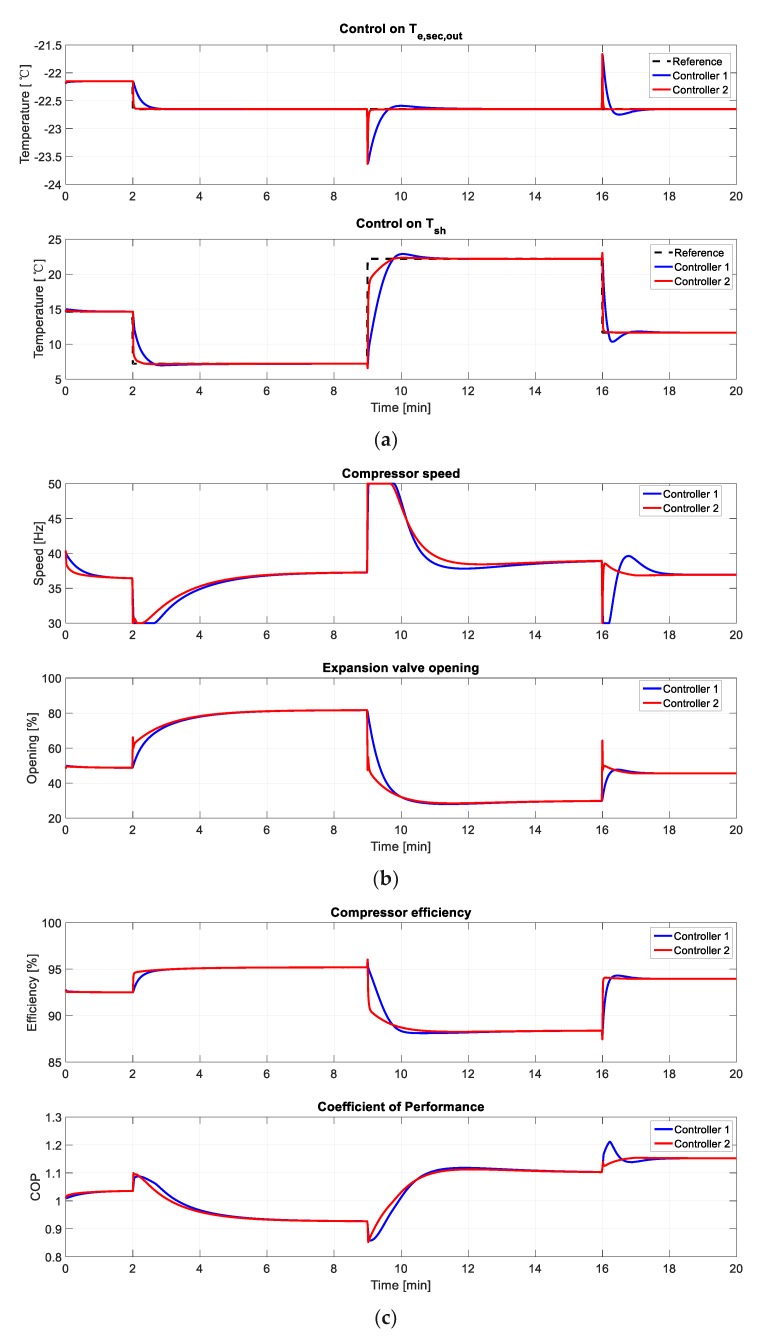
Simulation result of step response: (**a**) step response curves of *T_e,sec,out_* and *T_sh_*; (**b**) trend curves of *A_v_* and *N*; (**c**) trend curves of the compressor efficiency and COP.

During the step response process, *T_e,sec,out_* changes from −22.15 °C to −22.65 °C at 2 min, while *T_e,sec,out_* changes from 14.65 °C to 7.2 °C at 2 min, from 7.2 °C to 22.2 °C at 9 min, and from 22.2 °C to 11.65 °C at 16 min, respectively. Controller 1 represents the original controller, while controller 2 represents the optimal PID controller in Section 3.3. The simulation result is shown in Figure 11.

Table 2 displays the detailed data of the simulation:

### 4.2. Discussion

From the simulation result, we can tell that the optimal PID controller has a better control performance. The absolute value of the overshoot of the original controller varies from 0 to 11.24, while the overshoot of the optimal PID controller keeps at 0 for most of the conditions. Plus, the settling time of the optimal PID controller (4.20 s~120.18 s) is much shorter than the original controller (49.02 s~169.20 s). In Figure 11b, we can tell that the saturation time of the optimal PID controller is also much shorter than the original controller. In addition, in Figure 11c, the compressor efficiency and the coefficient of performance (COP) of the two controllers are about the same in the steady state, and the optimal PID controller obtains a faster dynamic response.

In conclusion, the proposed controller has the merits of less overshoot, less settling time, less saturation time, and faster dynamic response. As a result, the optimized controller is better than the original controller.

## 5. Conclusions

Refrigeration systems are critical for public health and carbon emissions. To deal with the characteristics of nonlinearity and high coupling, this paper solves this problem by applying an intelligent FOA algorithm to optimize the parameters of PID controllers to improve the control performance. This method not only is easy to code and realize, but also retains simplicity and reliability advantages of PID controllers. In this paper, a linear 2 × 2 MIMO system is identified based on the refrigeration system model, and FOA is employed to optimize the parameters of the PID controller for this system. The optimal PID control is finally put into use to control the nonlinear refrigeration system, and the simulation results illustrate that the optimal PID controller is able to regulate the overshoot, reduce the settling time, and restrain the controller saturation phenomenon. As a result, the optimal PID controller has a more accurate control performance and can help the refrigeration system emit less carbon dioxide during the process of dynamic regulation. With these advantages, the optimal PID controller based on FOA is both good for the environment, and public health. Therefore, it can be an ideal controller for refrigeration systems

## Figures and Tables

**Table 1:** Variables in the refrigeration system.

Variable	Description	Range	Initial Value	Units
*A_v_*	The valve opening	[10~100]	50	%
*N*	The compressor speed	[30~50]	40	Hz
*T_c,sec,in_*	Inlet temperature of the condenser secondary flux	[27~33]	30	°C
${\dot{m}}_{c,sec}$	Mass flow of the condenser secondary flux	[125~175]	150	g·s^−1^
*P_c,sec,in_*	Inlet pressure of the condenser secondary flux	--	1	bar
*T_e,sec,in_*	Inlet temperature of the evaporator secondary flux	[−22~−18]	−20	°C
${\dot{m}}_{e,sec}$	Mass flow of the evaporator secondary flux	[0.0075~0.055]	64.503	g·s^−1^
*P_e,sec,in_*	Inlet pressure of the evaporator secondary flux	--	1	bar
*T_surr_*	Compressor surroundings temperature	[20~30]	25	°C
*T_e,sec,out_*	The outlet temperature of the evaporator secondary flux	[−22.1~−22.6]	−22.1	°C
*T_sh_*	The degree of superheating	[7.2~22.2]	14.65	°C

**Table 2:** (**a**) Detailed data of the simulation result of *T_e,sec,out_*; (**b**) Detailed data of the simulation result of *T_sh_*.

(**a**)				
	**Controller 1**		**Controller 2**	
**Number**	**Overshoot (%)**	**Settling Time (s)**	**Overshoot (%)**	**Settling Time (s)**
1	0	49.02	0	19.02
2	−0.26	169.20	0	19.98
3	0.44	91.21	0	4.20
(**b**)				
	**Controller 1**		**Controller 2**	
**Number**	**Overshoot (%)**	**Settling Time (s)**	**Overshoot (%)**	**Settling Time (s)**
1	−3.14	112.23	0	22.98
2	3.11	150.04	0.68	120.18
3	−11.24	139.20	0	43.01
